# SLC39A8/Zinc Suppresses the Progression of Clear Cell Renal Cell Carcinoma

**DOI:** 10.3389/fonc.2021.651921

**Published:** 2021-03-25

**Authors:** Lilong Liu, Yaxin Hou, Junyi Hu, Lijie Zhou, Ke Chen, Xiong Yang, Zhengshuai Song

**Affiliations:** ^1^Department of Urology, Union Hospital, Tongji Medical College, Huazhong University of Science and Technology, Wuhan, China; ^2^Department of Urology, The Central Hospital of Wuhan, Tongji Medical College, Huazhong University of Science and Technology, Wuhan, China

**Keywords:** ccRCC, bioinformatics analysis, SLC39A8, zinc, progression

## Abstract

Clear cell renal cell carcinoma (ccRCC) is the most frequent and lethal subtype, which has high risk of metastasis or recurrence, accounting for 75–83% of renal cell carcinoma (RCC). Zrt‐ and Irt‐like proteins (ZIP) family members (SLC39A1-14) function to pass zinc into the cytoplasm for many critical biological processes when cellular zinc is depleted. However, the functional analysis of individual ZIP family genes in ccRCC is not clarified. This study aimed to investigate whether ZIP family genes are related to the clinicopathological features and survival of ccRCC patients, and to identify the function of key gene of ZIP family in ccRCC *in vitro*. Through bioinformatics analysis of tumor databases, SLC39A8 was identified as a key gene of ZIP family in ccRCC, which could be used as an effective indicator for diagnosing ccRCC and judging its prognosis. With the progression of tumor, the expression of SLC39A8 decreased progressively. The prognosis of patients with low expression of SLC39A8 is significantly worse. Furthermore, we found that overexpression of SLC39A8 or treatment with low concentration of zinc chloride could effectively inhibit the proliferation, migration and invasion of ccRCC cells. Moreover, the inhibition effect of SLC39A8 overexpression could be enhanced by low concentration zinc supplement. Therefore, this study provides a novel understanding for the role of SLC39A8/zinc in the regulation of ccRCC progression. These findings provide a new direction and target for progressive ccRCC drug development and combination therapy strategies.

## Introduction

The annual incidence rate of kidney cancer in the European Union reached 3.3% leading to approximately 99,200 new cases and 39,100 related deaths in 2018 (1). Renal cell carcinoma (RCC) is one of the most common malignant tumor of renal tubular epithelial cells origin, accounting for 5 and 3% of malignant tumors of males and females (2), and accounts for >90% of kidney cancer cases (3). The classic presentation of RCC includes low backache, hematuria, and a palpable abdominal mass. But not many patients now present in this manner. About half of the cases are now detected because a kidney mass is accidentally identified during the radiological examination (4). Due to the difficulty of early diagnosis of RCC, around one-third of patients present with metastatic disease at the time of diagnosis (5, 6). Moreover, those with nonmetastatic localized tumors have up to 40% risk of recurrence (5) and 21% risk of metastasis (7) following complete resection. The pathological types of clear cell renal cell carcinoma (ccRCC) is the most frequent and lethal subtype, which has high risk of metastasis or recurrence (8), accounting for 75–83% of RCC (3, 8–10). Currently, targeted therapies have been made in the treatment of ccRCC, including kinase and immune checkpoint inhibitors, which have greatly improved the treatment (11–14). However, there are still some dilemmas waiting to be solved, for example, all the patients do not gain from the treatment, development of drug resistance, loss of efficacy of a particular agent, etc. (11, 13). Hence, it is necessary to further elucidate the molecular mechanisms associated with ccRCC progression and metastasis, contributing to the development of novel therapeutic strategies.

As an essential micronutrient, zinc is the second richest and essential trace element in human body after iron (15), and approximately 98% is localized in the intracellular compartment (16). Most of the intracellular zinc is bound to or at least associated with proteins or complexed by anions (17). As previous studies reported, there were 2,800 human proteins potentially zinc-binding *in vivo*, corresponding to 10% of the human proteome (18). Zinc plays a critical and unique role in many critical biological processes including regulation of gene expression, DNA repair, antioxidant defense, enzyme function, immune function, endocrine function, growth, development, reproduction, and cancer biology (15, 16, 19). Prostate cancer cells have been found to have low levels of zinc, then lower zinc abolish mitochondrial aconitase inhibition, causing genetic/metabolic transformation (20). However, zinc could act as a tumor suppressor agent (20, 21), for example, the accumulation of zinc inhibits growth and proliferation of prostate cells (22), and zinc could induce apoptosis in prostate cancer cells through its direct effects on mitochondrial release of cytochrome C followed by activation of caspases-9 and -3 (23) and inhibition of nuclear factor kappa B (NF-kB) (24, 25). Notably, zinc could also inhibit the invasive and metastatic capabilities of prostate cancer cells (26–28). In addition, the regulation of zinc has also been reported to be associated with head and neck, esophageal, lung, pancreatic, prostate, and breast cancer (29–36).

The solute carrier 30 family (SLC30A/ZnT) and the Zrt‐ and Irt‐like proteins (ZIP/solute carrier 39 family, SLC39A) of zinc transporters are involved in controlling cellular zinc homeostasis in the body (37, 38). When the cellular zinc concentrations are elevated, ZnT family members function to the cytoplasmic zinc balance by exporting zinc out to the extracellular space or by isolating cytoplasmic zinc to the intracellular compartments (37). While ZIP family members serve to pass zinc into the cytoplasm when cellular zinc is depleted (38). The 14 human ZIP family proteins (SLC39A1, 2, 3, 4, 5, 6, 7, 8, 9, 10, 11, 12, 13, 14) are expressed in a wide variety of tissues and function in many different cellular processes (39). To the best of our knowledge, the functional analysis of individual ZIP family genes in patients with ccRCC is not clarified. This study aimed to investigate whether ZIP family genes are related to the clinicopathological features and survival of ccRCC patients. In addition, we also studied the function of key gene (SLC39A8) of ZIP family in ccRCC *in vitro*.

## Materials and Methods

### Cancer Database Bioinformatic Analysis

UALCAN, an interactive web-portal to perform to in-depth analyses of The Cancer Genome Atlas (TCGA) gene expression data (40), is publicly available at http://ualcan.path.uab.edu/index.html. This database was used to analyze relative expression of ZIP family genes across tumor and normal samples of ccRCC, as well as the tumor sub-groups based on individual tumor grade. GEPIA (Gene Expression Profiling Interactive Analysis) is a web-based tool to deliver fast and customizable functionalities based on TCGA and Genotype-Tissue Expression (GTEx) data, providing key interactive and customizable functions including differential expression analysis, profiling plotting, correlation analysis, patient survival analysis, similar gene detection and dimensionality reduction analysis (41), is publicly available at http://gepia.cancer-pku.cn/. We used this database to analyze the overall survival (OS) and disease-free survival (DFS) of ZIP family genes in ccRCC with the high and low groups cutoffs were 50% and 50%, the p value <0.05 was considered to have significant differences. Metascape combines functional enrichment, interactome analysis, gene annotation, and membership search to leverage over 40 independent knowledge bases within one integrated portal (42). Functional enrichment analysis of ZIP family genes was conducted with Metascape database (https://metascape.org/gp/index.html). The University of California, Santa Cruz (UCSC) Xena browser was developed as a high-performance visualization and analysis tool for both large public repositories and private datasets (43), available at https://xenabrowser.net/. We downloaded the mRNA expression level data of ZIP family genes in ccRCC tissues and corresponding normal tissues, the clinicopathological and survival data of ccRCC patients from the UCSC Xena browser. ONCOMINE, a cancer microarray database and web-based data-mining platform aimed at facilitating discovery from genome-wide expression analyses (44), is publicly available at https://www.oncomine.org/resource/main.html. Relative expression of SLC39A8 in tumor and normal samples of ccRCC in statistics by Beroukhim et al. (45), Jones et al. (46), Lenburg et al. (47), and Gumz et al. (48) were downloaded from ONCOMINE database. The E-MTAB-1980 cohort (https://www.ebi.ac.uk/arrayexpress/experiments/E-MTAB-1980/) with 101 sample information (49) was used as external validation to evaluate the clinicopathologic significance of SLC39A8 expression and OS in ccRCC patients. The Human Protein Atlas is a Swedish-based program aim to map all the human proteins in cells, tissues and organs using an integration of various omics technologies, including antibody-based imaging, mass spectrometry-based proteomics, transcriptomics and systems biology (50, 51), is publicly available at https://www.proteinatlas.org. We used this database to determine the expression of protein SLC39A8 in RCC and to account for the difference between tumor and normal samples through antibody-based imaging. Gene Set Enrichment Analysis (GSEA) is a powerful analytical method which derives its power by focusing on gene sets, that is, groups of genes that share common biological function, chromosomal location, or regulation (52). To determine which the hallmark effector gene sets associated with SLC39A8 mRNA expression of the TCGA-KIRC dataset, a GSEA was performed by GSEA software obtained from http://www.broad.mit.edu/gsea. For the enriched gene sets, the false discovery rate (FDR) value <0.25 and P <0.05 after performing 1,000 permutations were considered to be a statistically significant enrichment pathway. A Cancer Dependency Map was developed to identify genes essential for cancer cell proliferation/survival and facilitate the prioritization of therapeutic targets (53). Data evaluating the dependency of SLC39A8 gene for cell survival of renal cancer were downloaded from Depmap portal (https://depmap.org/portal). CERES Dependence Score of 0 and −1 represent the median scores of nonessential and cell-essential genes, respectively (54).

### Tissue Samples

Thirty-two pairs of T3 & T4 human ccRCC tissues and paired adjacent tissues from 32 ccRCC patients were collected by the Department of Urology, Union Hospital, Tongji Medical College (Wuhan, China). Among these, 24 pairs of tissue specimens were preserved in liquid nitrogen for subsequent protein expression analysis by western blotting and mRNA expression analysis by quantitative real-time PCR (qRT-PCR). The remaining eight pairs of samples were fixed with 4% paraformaldehyde for 24–48 h at room temperature and embedded in paraffin after dehydration and transparency for subsequent immunohistochemical (IHC) analysis. This study was fully informed by the patients and was approved by the Human Research Ethics Committee of Huazhong University of science and Technology (Wuhan, China).

### Cell Lines and Cell Culture

The normal human renal epithelial cell line HK2 and the RCC cell lines 786-O, OSRC-2, ACHN, A498 and CAKI-1 were purchased from the American Type Culture Collection (ATCC). All the cell lines were cultured in high glucose Dulbecco’s Modified Eagle’s medium (DMEM, Servicebio Co., Ltd., China) containing 10% fetal bovine serum (FBS, Gibco, USA) and 1% penicillin–streptomycin (Beijing Solarbio Science & Technology Co., Ltd., China). As for zinc supplementation experiment, the culture medium was replaced with new culture medium containing different concentrations of zinc chloride (a final concentration of 0, 0.5, 5, 10, 20, 50, 75, 100, 150, 200 µM) after the cells adhered completely. All cells were maintained in a cell incubator with 5% CO2, humidified and 37°C atmosphere (Thermo Fisher Scientific, Inc., USA).

### Cell Transfection

Small interfering RNA (siRNA) specifically targeting SLC39A8 (si−SLC39A8) and corresponding negative control siRNA (si-NC) (LOT. NO. R008263554), and the plasmids harboring SLC39A8 (SLC39A8) and a negative control (SLC39A8-NC) were constructed and supplied by Vigene Biology (Vigene, China). 786-O and OSRC-2 cells were transfected by si−SLC39A8, si-NC, SLC39A8, and SLC39A8-NC with lipofectamine^®^ 2000 (Thermo Fisher Scientific, Inc., USA) according to the manufacturer’s instructions. Cells were collected 48 h after transfection for subsequent experiments. The si-SLC39A8 sequence was as follows: 5’-CCUUGUAUGCAGGAGAAAUTT-3’.

### Immunohistochemistry

Eight pairs of tissue specimens from ccRCC patients were cut into 5 μm paraffin-embedded sections. The tissue sections were incubated with SLC39A8 rabbit polyclonal antibody (1:200, ABclonal, China) overnight at 4°C, then the immunodetection was performed using secondary antibody at room temperature. Next, the results were visualized by 3,3’-diaminobenzidine and hematoxylin. Finally, the prepared slides were scanned as high-resolution digital images using the Pannoramic MIDI II (3Dhistech, Hungary) histological scanner.

### Western Blotting Experiments

The cell lines and tissue samples protein were extracted by RIPA buffer (Servicebio Co., Ltd., China) containing proteinase inhibitor cocktail and phenylmethanesulfonyl fluoride (PMSF). An equal amount of the above products (total protein) was used for gel electrophoresis and transferred onto nitrocellulose membranes. Then, the membranes were incubated with primary antibodies overnight at 4°C after blocking with 5% milk for 1.5 h. Subsequently, the membranes were incubated with secondary antibodies at room temperature for 1.5 h and the proteins were visualized by ChemiDoc-XRs+ (Bio-Rad Laboratories, Inc., USA). Primary antibodies: GAPDH (1:3,000, Wuhan Boster Biological Technology, Ltd., China), SLC39A8 (1:1,500, ABclonal, China), N-cadherin (1:5,000, Abcam, USA), E-cadherin (1:10,000, Abcam, USA), SNAI1 (1:1,000, Bio-Swamp Life Science Lab, China). All the procedures were carried out according to the manufacturer’s instructions.

### RNA Extraction and qRT-PCR

The cell lines and tissue samples total mRNA was extracted by Trizol reagent (Thermo, USA), and the purity and concentration were tested by the NanoDrop 2000 spectrophotometer (NanoDrop Technologies, USA). About 2 μg of the above products were reversely transcribed to complementary DNA (cDNA) according to the manufacturer’s instructions. qRT-PCR was carried out using SYBR Green qPCR Master Mix (Vazyme Biotech, China) according to the manufacturer’s instructions. Each sample was repeated in triplicate. The relative gene expression level was expressed by the comparative CT method. Specific primers, SLC39A8 (Forward: 5’-TGGTTGCACCCCTCACAAAT-3’, Reverse: 5’-CACATGGTGCACTGAAACCG-3’), GAPDH (Forward: 5’-TCGTGGAAGGACTCATGACC-3’, Reverse: 5’-CCAGTGAGCTTCCCGTTCA-3’).

### Colony Formation Assay

786-O, OSRC-2 cells and 786-O, OSRC-2 cells transfected with si−SLC39A8, si-NC, SLC39A8, and SLC39A8-NC were seeded into 6-well plates (12-well plates for 786-O and OSRC-2 cells) with 1,000 cells per well. After the cells adhered completely, the culture medium was replaced with new culture medium containing different concentrations of zinc chloride. The cell colonies were fixed by methanol for 12 min and then stained with 0.05% crystal violet dye for 15 min after incubated for 12 days (9 days for 12-well plates).

### Cell Proliferation Assay

786-O, OSRC-2 cells and 786-O, OSRC-2 cells transfected with si−SLC39A8, si-NC, SLC39A8, and SLC39A8-NC were inoculated on 96-well plates with 1,000 cells per well. A cell proliferation assay was carried out per 24 h for a total of 96 h by using a Cell Counting Kit-8 (CCK8, MedChemExpress, USA) according to the manufacturer’s instructions. After incubation for 2 h in a cell incubator (Thermo Fisher Scientific, Inc., USA), the absorbance of each well was measured at 450 nm by a spectrophotometer to evaluate the quantity of living cells.

### Transwell Migration and Invasion Experiments

786-O and OSRC-2 cells transfected with si−SLC39A8, si-NC, SLC39A8 (overexpression), and SLC39A8-NC were used for subsequent experiments. Transwell chambers with 8−µm membrane filters and 24-well plates (Corning Inc., USA) were used in the migration and invasion assays. The cells were incubated in DMEM without FBS for 6–8 h, and then collected the cells for later use. For migration assay, 1 × 10^5^ cells in 200 µl FBS-free DMEM were seeded into the upper chambers, and the lower chambers were filled with 600 µl DMEM containing 10% FBS. For invasion assay, 2 × 10^5^ cells in 200 µl FBS-free DMEM were seeded into the upper chambers which had been coated with matrigel and incubated at 37°C for 6–8 h, and the lower chambers were also filled with 600 µl DMEM containing 10% FBS. Each group was tested in three replicates. After incubation at a cell incubator (Thermo Fisher Scientific, Inc., USA) with 5% CO_2_, humidified and 37°C atmosphere, the transwell chambers were transferred to a new 24-well plate, the upper and lower chambers were rinsed with phosphate buffer saline (PBS) twice, then the cells were fixed with 100% methanol for 15 min and stained with 0.05% crystal violet dye for 30 min at room temperature. Finally, the transwell chambers were rinsed in PBS for three times, the cells in the upper chamber were swabbed, and the cells in lower chambers were observed with a light microscope (Nanjing Jiangnan Novel Optics Co., Ltd., China) at 200× magnification, four microscopic fields were randomly selected for cell counting.

### Statistics

Graphpad Prism 6.0 and SPSS statistics software 21.0 were used for statistical analysis. The univariate and multivariate analyses of OS and DFS were performed to further screen the key genes of ZIP family in ccRCC patients. The SLC39A8 mRNA levels were analyzed in different clinicopathological parameters of ccRCC by Student’s t-test. The survival curve analysis was used to analyze the relationship between the expression level of SLC39A8 and OS or disease specific survival (DSS) of ccRCC patients. The receiver operating characteristic (ROC) curve was used to analyze the expression level of SLC39A8 to distinguish ccRCC patients and obtain the area under the curve. Rank sum test of ordered data was used to analyze the expression of protein SLC39A8 in RCC through antibody-based imaging from The Human Protein Atlas. Each group of data was presented as mean ± standard deviation (SD). Mean differences were considered statistically significant when P <0.05. *P <0.05, **P <0.01, ***P <0.001, ****P <0.0001.

## Results

### The Relative mRNA Expression of ZIP Family Genes and Their Prognostic Values in ccRCC

**Figure 1:**
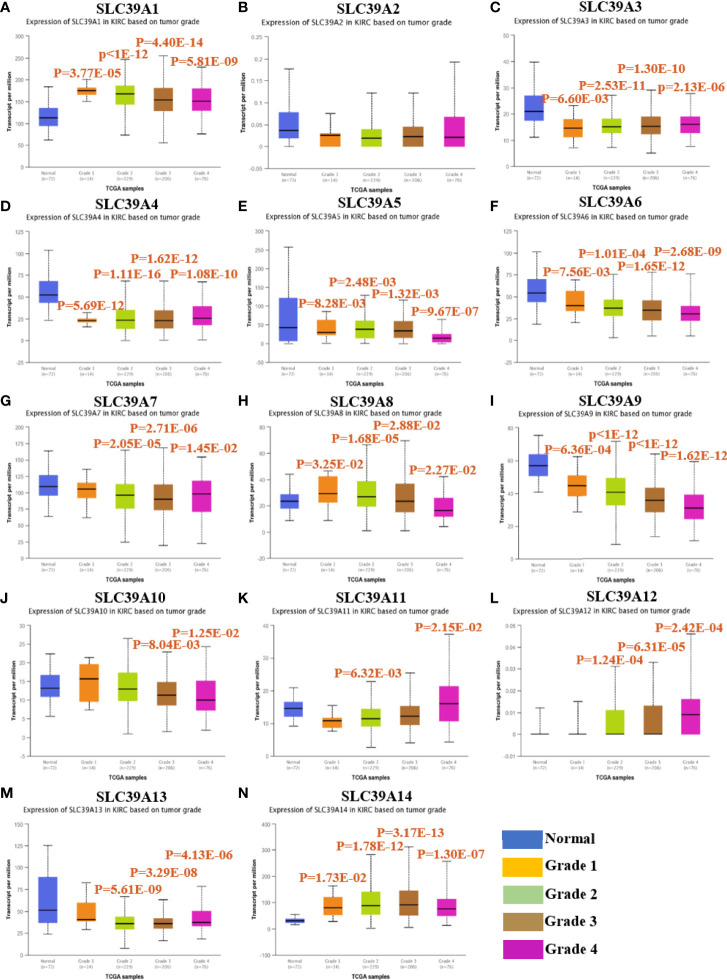
The expression of ZIP family genes based on tumor grade in patients with ccRCC by using UALCAN database^40^. **(A**–**N)** SLC39A1, SLC39A2, SLC39A3, SLC39A4, SLC39A5, SLC39A6, SLC39A7, SLC39A8, SLC39A9, SLC39A10, SLC39A11, SLC39A12, SLC39A13, SLC39A14. Mean differences were considered statistically significant when P <0.05.

**Figure 2:**
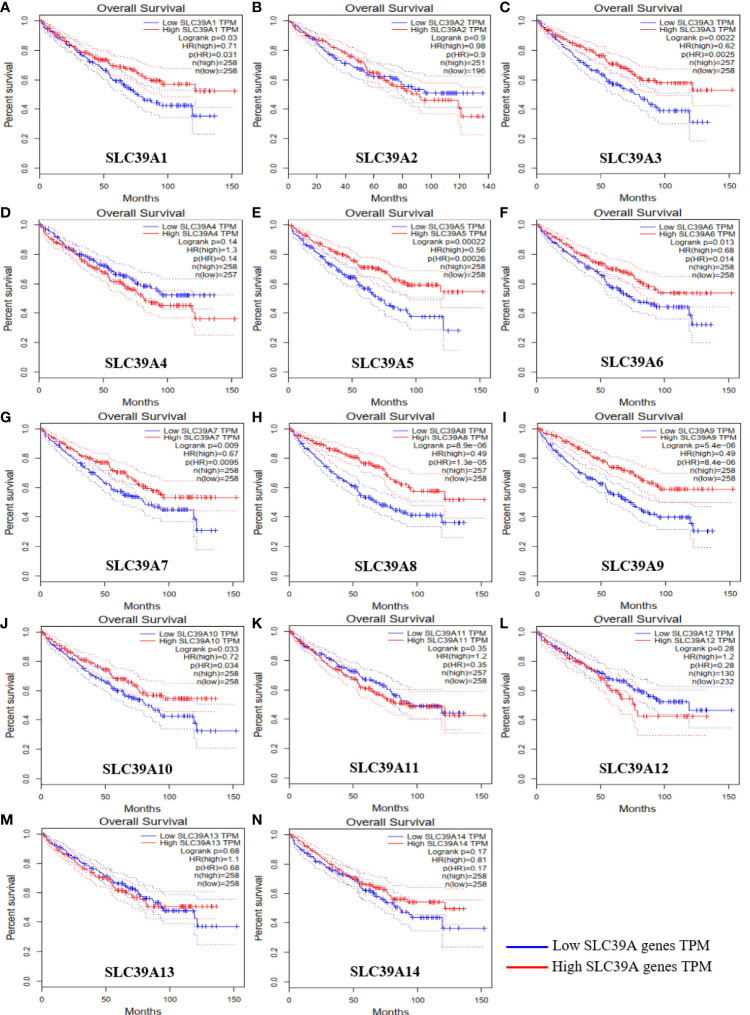
Overall survival of ZIP family genes in patients with ccRCC by using GEPIA. **(A**–**N)** SLC39A1, SLC39A2, SLC39A3, SLC39A4, SLC39A5, SLC39A6, SLC39A7, SLC39A8, SLC39A9, SLC39A10, SLC39A11, SLC39A12, SLC39A13, SLC39A14. Mean differences were considered statistically significant when P <0.05.

To explore the roles of ZIP family genes expression in ccRCC, the expression data from TCGA database were analyzed by using UALCAN. As shown in **Supplementary Figures 1A–N**, the expression of SLC39A1, SLC39A8, SLC39A12 and SLC39A14 in cancer tissues was significantly higher than that in normal tissues, however, the SLC39A3, SLC39A4, SLC39A5, SLC39A6, SLC39A7, SLC39A9 and SLC39A13 were expressed higher in normal tissues, and the expression of SLC39A2, SLC39A10 and SLC39A11 showed no significant difference between cancer and normal tissues. Further, the expression of ZIP family genes based on tumor grade was analyzed. As shown in **Figures 1A–N**, the expression of ZIP family genes was significantly different between normal tissues and cancer tissues of different tumor grades except SLC39A2. In addition, the potential prognostic values of ZIP family genes in ccRCC were investigated by using GEPIA. As shown in **Figures 2A–N**, SLC39A1, SLC39A3, SLC39A5, SLC39A6, SLC39A7, SLC39A8, SLC39A9, and SLC39A10 showed positive relationships between low expression and significant worse OS in patients with ccRCC. However, only SLC39A5, SLC39A8, and SLC39A9 showed positive relationships between low expression and significant worse DFS (**Supplementary Figures 2A–N**). Then, univariate and multivariate analyses of OS and DFS were performed to further find the key gene of ZIP family in ccRCC patients. The results indicated that only SLC39A8 regarded as prognostic factor in both OS and DFS (**Tables 1**, **2**). Thus, we identified SLC39A8 as a key gene of ZIP family in ccRCC.

**Table 1:** Univariate and multivariate analyses of ZIP family genes mRNA level and patient overall survival.

Variable	Univariate analysis			Multivariate analysis		
	HR	95%CI	P-value	HR	95%CI	P-value
SLC39A1	1.147	0.852–1.544	0.367			
SLC39A2	1.296	0.962–1.745	0.088			
SLC39A3	1.236	0.917–1.665	0.164			
SLC39A4	1.112	0.827–1.496	0.483			
SLC39A5	0.581	0.429–0.785	0.000	0.677	0.431–1.062	0.090
SLC39A6	0.885	0.658–1.191	0.420			
SLC39A7	1.159	0.862–1.559	0.329			
SLC39A8	0.600	0.443–0.814	0.001	0.586	0.367–0.935	0.025
SLC39A9	0.476	0.350–0.648	0.000	0.986	0.617–1.575	0.953
SLC39A10	0.698	0.518–0.942	0.019	1.046	0.655–1.670	0.850
SLC39A11	1.524	1.126–2.063	0.006	0.669	0.423–1.058	0.086
SLC39A12	1.202	0.891–1.622	0.228			
SLC39A13	1.820	1.342–2.467	0.000	1.520	0.947–2.441	0.083
SLC39A14	1.038	0.772–1.397	0.803			
Age	1.771	1.305–2.405	0.000	1.748	1.128–2.707	0.012
Gender	0.949	0.698–1.292	0.741			
T stage	1.915	1.628–2.254	0.000	0.935	0.560–1.562	0.797
N stage	3.392	1.801–6.389	0.000	1.383	0.653–2.928	0.397
M stage	4.351	3.190–5.935	0.000	1.705	0.751–3.873	0.202
TNM stage	1.882	1.651–2.145	0.000	1.393	0.810–2.395	0.230
Grade	2.286	1.868–2.796	0.000	1.338	0.946–1.893	0.100
Recurrence	2.281	0.666–7.816	0.189			

HR, hazard ratio; CI, confidence interval; TNM, Tumor Node Metastasis. Mean differences were considered statistically significant when P <0.05.

**Table 2:** Univariate and multivariate analyses of ZIP family genes mRNA level and patient disease-free survival.

Variable	Univariate analysis			Multivariate analysis		
	HR	95%CI	P-value	HR	95%CI	P-value
SLC39A1	1.422	0.995–2.033	0.053			
SLC39A2	1.334	0.936–1.901	0.111			
SLC39A3	1.299	0.912–1.851	0.147			
SLC39A4	1.011	0.711–1.439	0.950			
SLC39A5	0.593	0.415–0.848	0.004	0.503	0.302–0.838	0.008
SLC39A6	1.071	0.752–1.525	0.705			
SLC39A7	1.119	0.786–1.593	0.532			
SLC39A8	0.406	0.278–0.594	0.000	0.481	0.275–0.841	0.010
SLC39A9	0.481	0.335–0.691	0.000	0.810	0.485–1.353	0.421
SLC39A10	0.777	0.546–1.106	0.161			
SLC39A11	1.928	1.338–2.776	0.000	0.716	0.399–1.285	0.263
SLC39A12	0.855	0.592–1.234	0.403			
SLC39A13	1.790	1.252–2.559	0.001	1.037	0.609–1.765	0.895
SLC39A14	1.573	1.095–2.259	0.014	1.326	0.769–2.287	0.310
Age	1.343	0.945–1.911	0.100			
Gender	1.389	0.939–2.056	0.100			
T stage	2.517	2.043–3.102	0.000	0.805	0.434–1.494	0.493
N stage	4.690	2.293–9.592	0.000	1.901	0.857–4.218	0.114
M stage	8.595	5.918–12.483	0.000	1.340	0.519–3.460	0.545
TNM stage	2.678	2.247–3.191	0.000	2.391	1.215–4.706	0.012
Grade	3.026	2.345–3.904	0.000	1.319	0.907–1.918	0.148

HR, hazard ratio; CI, confidence interval; TNM, Tumor Node Metastasis. Mean differences were considered statistically significant when P <0.05.

### Relationship Between the mRNA Levels of SLC39A8 and the Clinicopathological Parameters of Patients With ccRCC

**Figure 3:**
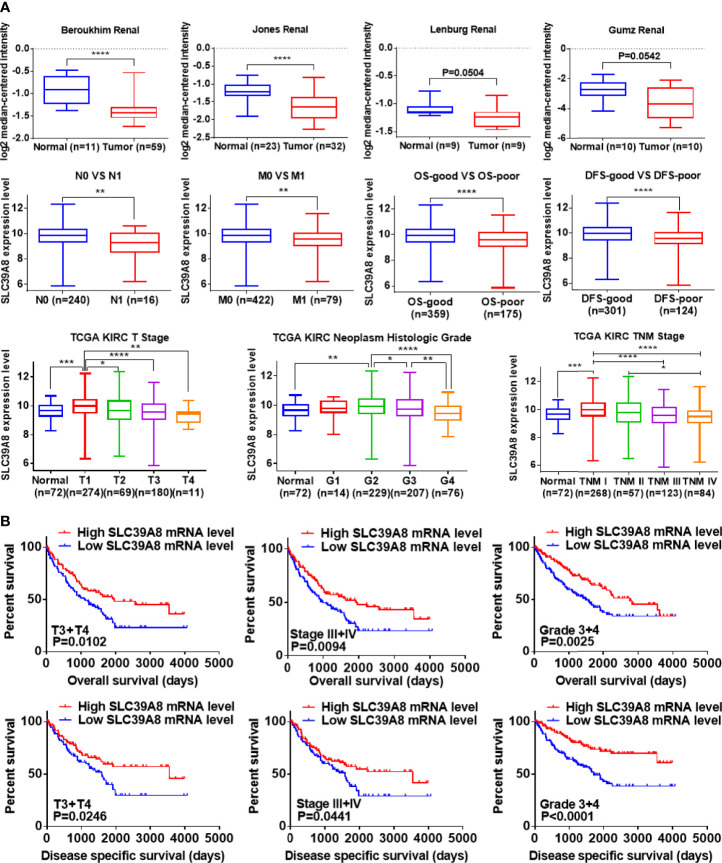
SLC39A8 was down expression and positively associated with tumor progression and worse prognosis in ccRCC patients. **(A)** ONCOMINE database analysis showed that the mRNA expression levels of SLC39A8 were down-regulated in ccRCC in statistics by Beroukhim et al. (45) and Jones et al. (46), but similar in two studies of Lenburg et al. (47) and Gumz et al. (48). In addition, data analysis based on TCGA showed that with the progression of tumor, the expression of SLC39A8 decreased progressively. **(B)** Survival curve analysis determined that low SLC39A8 expression exhibits a shorter OS and DSS time in progressive ccRCC patients (T3 + T4, Stage III + IV, Grade 3 + 4). OS, overall survival; DSS, disease specific survival. *P <0.05, **P <0.01, ***P <0.001, ****P <0.0001.

The ONCOMINE and TCGA databases were used to analyze the sequencing data of SLC39A8 in ccRCC. As shown in **Figure 3A**, the expression level of SLC39A8 was significantly lower in cancer tissues than that in normal tissues of ccRCC in statistics by Beroukhim et al. (45), Jones et al. (46), but similar in two studies of Lenburg et al. (47), and Gumz et al. (48). Next, the expression level of SLC39A8 in various subgroups of patients with ccRCC was evaluated. The results indicated that the expression of SLC39A8 in patients with distant metastasis or lymph node metastasis was significantly decreased, and the prognosis of those patients with decreased expression of SLC39A8 was significantly worse. Interestingly, the data from TCGA databases showed that the reduction of SLC39A8 expression was mainly occurred in patients with advanced ccRCC, while the expression level in patients with early ccRCC remained unchanged, even slightly increased. Notably, the survival curve analysis determined low SLC39A8 expression patients exhibited a shorter OS and DSS time (**Figure 3B**). In addition, the E-MTAB-1980 cohort was used to check the clinicopathologic significance of SLC39A8 expression, as shown in **Supplementary Figures 3A–E**, the expression of SLC39A8 was decreased in ccRCC patients with distant or lymph node metastasis, and decreased progressively with the increase of T stage and neoplasm histologic grade. Concordant with the results of TCGA database analysis, ccRCC patients with low expression of SLC39A8 had a poor prognosis. These results indicated that SLC39A8 is down-regulated in ccRCC and positively correlated with tumor progression.

To probe the diagnostic significance of low expression of SLC39A8 in ccRCC patients, we analyzed the diagnostic value of SLC39A8 low expression in TCGA data set in various clinicopathological parameters by ROC curve. As shown in **Supplementary Figure 4A**, ROC curve analysis showed that the expression level of SLC39A8 could statistically distinguish ccRCC from normal tissues with an AUC of 0.6378 (P = 0.0043). In addition, we also analyzed the diagnostic value of SLC39A8 expression level in subgroups as follows: N0 vs. N1 stage (AUC = 0.6767, P = 0.0180); M0 vs. M1 stage (AUC=0.6192, P=0.0008); T1 + T2 vs. T3 + T4 stage (AUC = 0.6390, P <0.0001); Stage 1 + Stage 2 vs. Stage 3 + Stage 4 (AUC = 0.6492, P <0.0001); Grade 1 + Grade 2 vs. Grade 3 + Grade 4 stage (AUC = 5879, P <0.0005); OS-good vs. OS-poor (AUC = 0.6209, P <0.0001); and DFS-good vs. DFS-poor (AUC = 0.6491, P <0.0001). These results suggest that SLC39A8 expression level has diagnostic value for ccRCC patients.

### SLC39A8 Expression in ccRCC Tissues and RCC Cell Lines

**Figure 4:**
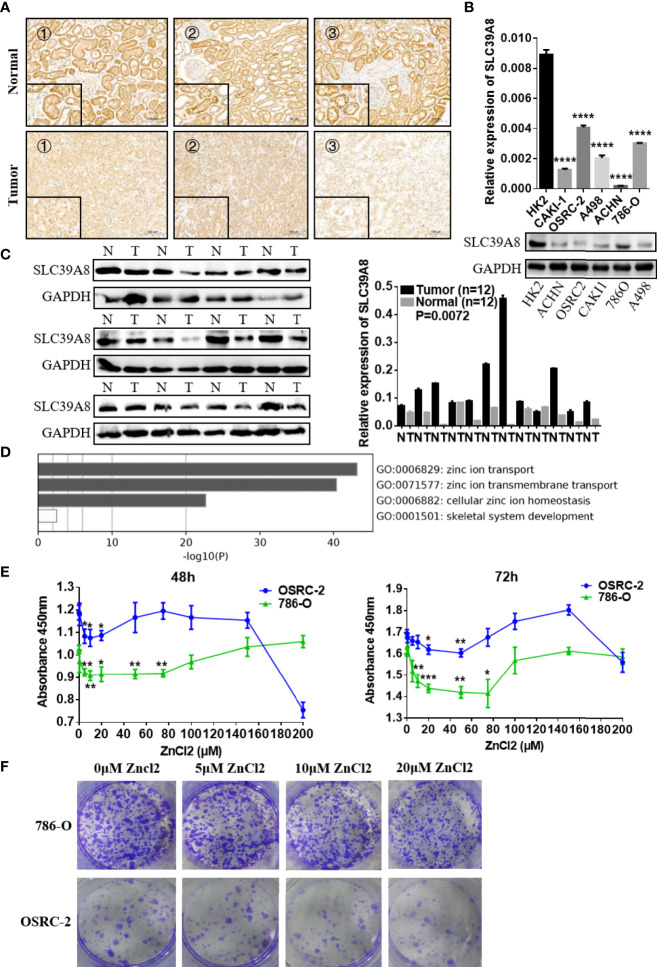
Function of SLC39A8 and its expression in ccRCC samples and RCC cell lines. **(A)** Immunohistochemistry for SLC39A8 expression in ccRCC tissues and the corresponding normal tissues. The inserted images are the higher magnification picture of a certain part of the same tissue, ×100 and ×400. **(B, C)** Reverse transcription-quantitative PCR assays and western blotting experiments of SLC39A8 expression in normal human renal epithelial cell line HK2 and RCC cell lines (786-O, OSRC-2, ACHN, A498 and CAKI-1) and ccRCC patients. **(D)** Functional enrichment analysis of ZIP family genes by Metascape. The results showed that ZIP family functions in the cellular import of zinc. **(E)** Cell proliferation assay of 786-O and OSRC-2 cells treated with different concentrations of zinc chloride (0–200 μM) for 48–72 h. **(F)** The colony formation assay of 786-O and OSRC-2 cells treated with 0–20 μM zinc chloride. The values of each group were presented as the mean ± standard deviation. *P <0.05, **P < 0.01, ***P < 0.001, ****P < 0.0001.

As SLC39A8 was down-regulated in the TCGA data set, immunohistochemistry, qRT-PCR, and western blotting experiments were performed to verify the expression levels of SLC39A8 in ccRCC tissues and RCC cell lines. As shown in **Figure 4A**, SLC39A8 protein was mainly located in the plasma membrane of renal tubular epithelial cells from paracancer tissues and cancer cells, the SLC39A8 expression in ccRCC tissues was significantly down-regulated. Moreover, we investigated the expression of protein SLC39A8 in RCC using online database The Human Protein Atlas, the results also indicated that SLC39A8 was down-regulated in RCC (**Supplementary Figure 4B**). At the cell line level, we also found that the expression of SLC39A8 in RCC cell lines was significantly lower than that in HK2 by qRT-PCR and western blotting experiments (**Figure 4B**). In addition, we confirmed SLC39A8 expression in T3 & T4 human ccRCC tissues and paired adjacent tissues by qRT‐PCR and western blotting experiments (**Figure 4C**). All the results demonstrated that SLC39A8 expression level was significantly lower in RCC cell lines and T3 & T4 ccRCC tissues.

### The Effect of Zinc Supplementation on Cell Proliferation of ccRCC Cells

Functional enrichment analysis of ZIP family genes was conducted with Metascape. The results showed that ZIP family functions in the cellular import of zinc (**Figure 4D**). The CERES dependence score obtained from Depmap portal showed that SLC39A8 is not a key gene for RCC cells survival (**Supplementary Figure 4C**). Considering that the expression of SLC39A8 is down-regulated in ccRCC cells and SLC39A8 is involved in zinc transport, we speculated that ccRCC cells are in a low intracellular zinc state. Thus, we tried to treat ccRCC cell lines with different concentrations of zinc chloride, and surprisingly, we found that low concentrations (5–75 µM) of zinc supplementation for 48–72 h could significantly inhibit the 786-O and OSRC-2 cells proliferation (**Figure 4E**). The colony formation assay also showed that 10 and 20 µM zinc chloride treatment could significantly inhibit the colonies formation of 786-O and OSRC-2 cells (**Figure 4F**). Since 20 µM zinc treatment could significantly inhibit the growth and colony formation of 786-O and OSRC-2 cells, 20 µM zinc chloride was chosen for the subsequent experiments unless otherwise stated.

### Biological Function of SLC39A8/Zinc in ccRCC Cell Lines

**Figure 5:**
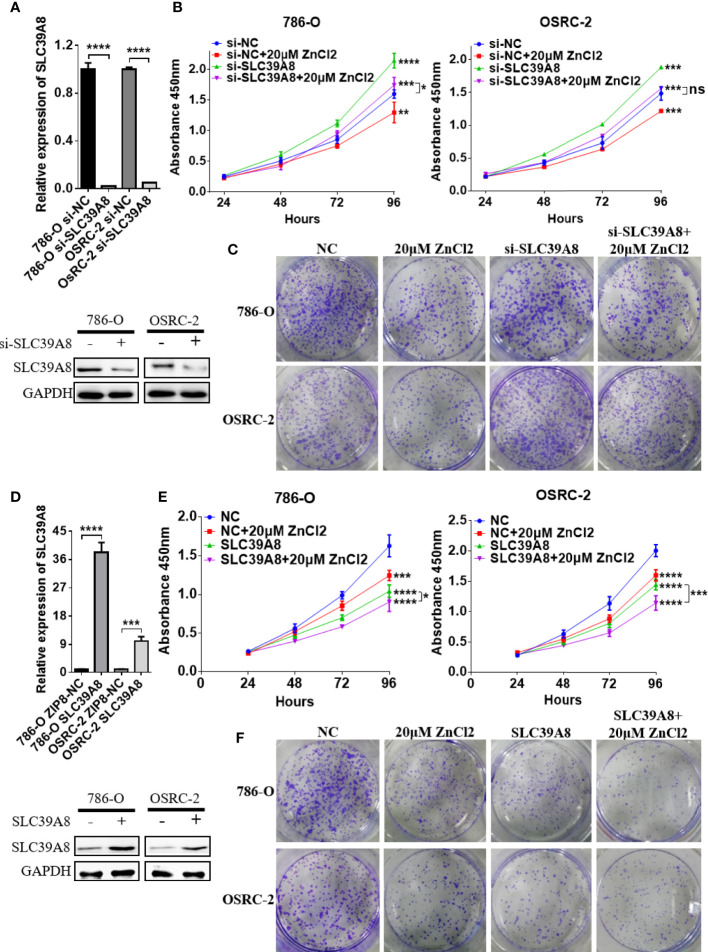
SLC39A8/zinc inhibits the proliferation of ccRCC cells *in vitro*. **(A)** Quantitative real-time PCR and western blotting experiments of SLC39A8 knockdown in 786-O and OSRC-2 cells. **(B, C)** Cell proliferation assay and colony formation assay detected the effects of SLC39A8 knockdown with or without zinc supplementation on the proliferation of 786-O and OSRC-2 cells. **(D)** Quantitative real-time PCR and western blotting experiments of SLC39A8 overexpression in 786-O and OSRC-2 cells. **(E, F)** Cell proliferation assay and colony formation assay detected the effects of SLC39A8 overexpression with or without zinc supplementation on the proliferation of 786-O and OSRC-2 cells. The values of each group were presented as the mean ± standard deviation. *P < 0.05, **P < 0.01, ***P < 0.001, ****P < 0.0001.

**Figure 6:**
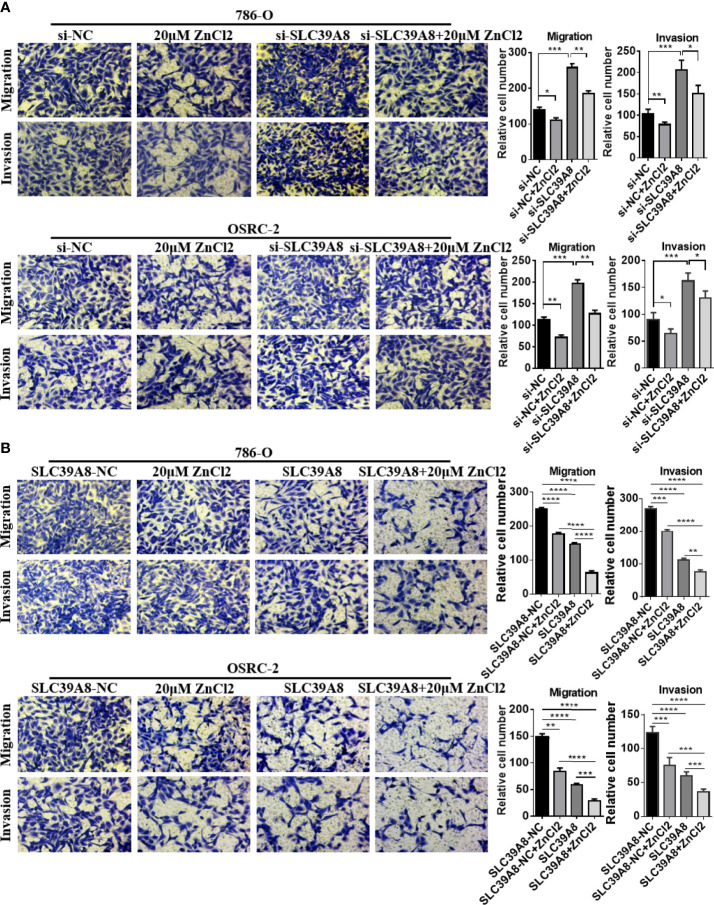
SLC39A8/zinc inhibits the invasion and migration of ccRCC cells *in vitro*. **(A)** Representative images of migration and invasion assays of SLC39A8 knockdown-786-O and OSRC-2 cells. **(B)** Representative images of migration and invasion assays of SLC39A8 overexpressed-786-O and OSRC-2 cells. Four microscopic fields with ×200 magnification were randomly selected for cell counting, and the data were presented as the mean ± standard deviation. *P < 0.05, **P < 0.01, ***P < 0.001, ****P < 0.0001.

To identify the function of SLC9A8 on the biological behaviors of ccRCC, 786-O and OSRC-2 cell lines were chosen to transfect with si−SLC39A8 to down-regulate the expression of SLC39A8. As shown in **Figure 5A**, SLC39A8 mRNA and protein expression levels were significantly decreased in 786-O & OSRC-2-si−SLC39A8 cells compared with the corresponding control. After transfection, cell proliferation assay and colony formation assay were performed. The results demonstrated that knocking down SLC39A8 could significantly promote cell proliferation, while zinc supplementation could reverse this phenomenon (**Figures 5B, C**). In order to verify this discovery, 786-O and OSRC-2 cells were transfected with SLC39A8 to up-regulate the expression of SLC39A8. As shown in **Figures 5D–F**, overexpression of SLC39A8 could significantly inhibit the proliferation of 786-O and OSRC-2 cells, and zinc supplementation could enhance the inhibitory effect. Next, we investigated the effect of SLC39A8/zinc on the invasion and migration of ccRCC cells. As shown in **Figure 6A**, knocking down SLC39A8 could significantly promote cell invasion and migration, while zinc supplementation could reverse this phenomenon to a certain extent. Conversely, overexpression of SLC39A8 could significantly inhibit cell invasion and migration, and zinc supplementation could enhance this inhibitory effect (**Figure 6B**). These results indicated that SLC39A8/zinc could inhibit the proliferation, migration and invasion of ccRCC cells.

### Mechanism of SLC39A8/Zinc Inhibiting Cell Migration and Invasion

**Figure 7:**
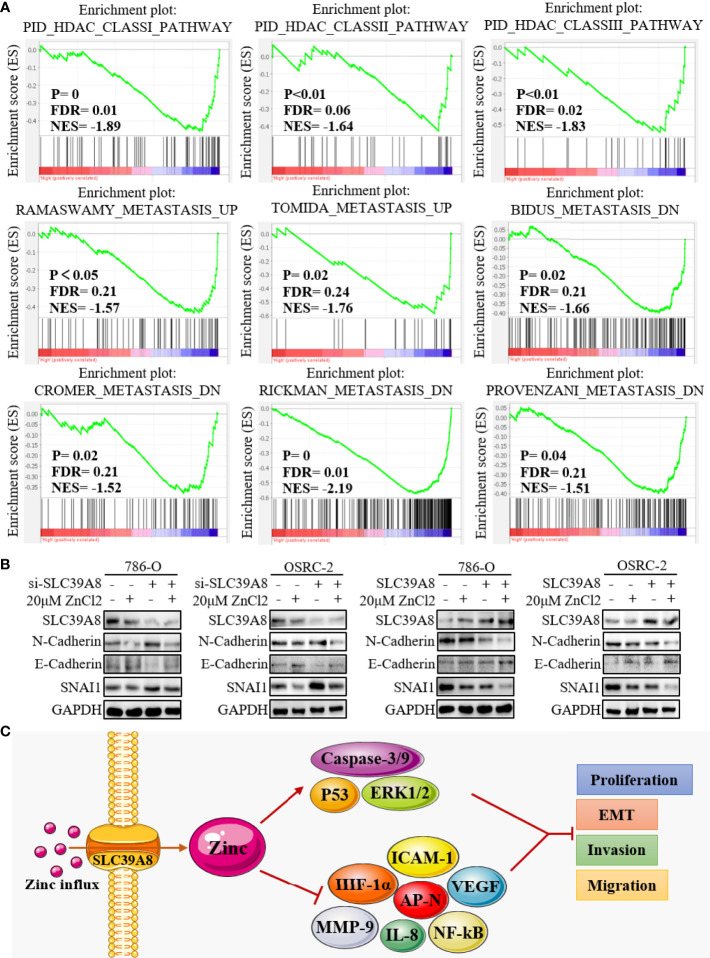
Mechanism of SLC39A8/zinc inhibiting cell migration and invasion. **(A)** GSEA showed that the group with low expression of SLC39A8 was significantly associated with HDAC and metastasis pathway. **(B)** Western blotting experiments detected the effect of SLC39A8/zinc on EMT in ccRCC cells. 786-O and OSRC-2 cells transfected with si−SLC39A8 (knockdown), SLC39A8 (overexpression), and their corresponding control were used for this study. **(C)** A possible mechanism for SLC39A8/zinc inhibits the EMT of ccRCC cells. HDAC, histone deacetylase; EMT, epithelial-mesenchymal transition; ICAM-1, intercellular adhesion molecule 1; AP-N, aminopeptidase N; NF-kB, nuclear factor kappa B; VEGF, vascular endothelial growth factor; IL-8, interleukin 8; MMP-9, matrix metallopeptidase 9; HIF-1α, hypoxia inducible factor-1α; ERK1/2, extracellular signal-regulated kinase 1/2.

GSEA was performed using TCGA database to obtain more information related to the biological pathways of ccRCC. The results showed that the group with low expression of SLC39A8 was significantly enriched in nine functional gene sets which associated with histone deacetylase (HDAC) or metastasis pathway (**Figure 7A**). Subsequently, we verified the effect of SLC39A8/zinc on epithelial–mesenchymal transition (EMT) in ccRCC cells. 786-O and OSRC-2 cells transfected with si−SLC39A8 (knockdown), SLC39A8 (overexpression), and their corresponding control were used for this study. As shown in **Figure 7B**, through knocking down SLC39A8, western blotting experiments confirmed the enhanced expression of interstitial marker N-Cadherin and transcription factor SNAI1, while the epithelial marker E-Cadherin expression was reduced in 786-O & OSRC-2-si−SLC39A8 cells. However, zinc supplementation reverses this change to some extent. On the other hand, up-regulated expression of E-Cadherin and down-regulated expression of N-Cadherin and SNAI1 were observed in 786-O & OSRC-2-SLC39A8 cells, and this effect could be enhanced by zinc supplementation. These results suggested that the low expression of SLC39A8 in 786-O and OSRC-2 cells induced transition to the interstitial phenotype, on the contrary, overexpression of SLC39A8 induced transition to the epithelial phenotype, and this stimulation was enhanced by low concentration zinc supplement.

## Discussion

Zinc plays a crucial role in various biological events, intracellular and extracellular zinc levels and distributions is critically regulated by two protein families of zinc transporters, ZnT family functions to export zinc out to the extracellular space (37), and ZIP family serves to pass zinc into the cytoplasm (38). It has been reported that abnormal zinc transporters are associated with many specific diseases, including Alzheimer’s disease, diabetes, and cancers (15, 55, 56). For the cancer, zinc generally acts as a suppressive agent (20, 21, 33), accumulating evidence has indicated that zinc deficiency contributed to increased cancer risk, tumor size, tumor stage, and increased unplanned hospitalizations by modified zinc homeostasis, transporter activity and affected ion channel activity (15, 57–59). However, high dietary zinc intake has been reported to decrease the risk of colon cancer (60). Based on these studies, we can know that the change of expression profile of ZIP family members will inevitably affect the intracellular and extracellular zinc levels, and then affect the occurrence and development of tumors.

In this study, based on TCGA database analysis, we found that the expression of SLC39A1, SLC39A8, SLC39A12 and SLC39A14 were significantly higher in cancer tissues compared to normal tissue, while the SLC39A3, SLC39A4, SLC39A5, SLC39A6, SLC39A7, SLC39A9 and SLC39A13 were expressed higher in normal tissues. However, the low expression of SLC39A1, SLC39A3, SLC39A5, SLC39A6, SLC39A7, SLC39A8, SLC39A9, and SLC39A10 implies a poor OS in patients with ccRCC. This contradiction could be clearly explained by the following analysis, that is, the expression of SLC39A8 is slightly up-regulated in early tumors, but with the increase of tumor grade, its expression level decreased gradually. This indicates that the down-regulation of SLC39A8 is involved in ccRCC progression. In addition, SLC39A8 was identified as a key functional gene of ZIP family in ccRCC by univariate and multivariate analyses. ROC curve analysis also proved that SLC39A8 could be used as an effective index to diagnose ccRCC and judge its prognosis. Considering that SLC39A8 is involved in passing zinc into the cytoplasm (38), as confirmed by functional enrichment analysis, we believe that the decrease of intracellular zinc promotes the progression of ccRCC.

Although multiple advances have been made in systemic therapy for RCC in recent years, metastatic RCC remains incurable (10). Among RCC, ccRCC is the most frequent and lethal subtype with high risk of metastasis and recurrence (8). Thus, effective treatment of progressive ccRCC is the most important part of the treatment of RCC. Previous studies have shown that lower zinc may promote proliferation and inhibit apoptosis of prostate cells (22–25), while zinc supplementation could suppress its EMT through inhibiting intercellular adhesion molecule 1 (ICAM-1) expression and aminopeptidase N (AP-N) activity (27, 28, 33). In addition, zinc sulphate treatment could also reduce the expression of some other angiogenic and metastatic factors, including vascular endothelial growth factor (VEGF), interleukin 8 (IL-8) and matrix metallopeptidase 9 (MMP-9) (27). Moreover, a latest study showed that zinc may inhibit cell proliferation of esophageal cancer cells through Orai1 (a store-operated Ca^2+^ entry channel)-mediated intracellular Ca^2+^ oscillations and revealed a possible molecular basis for zinc-induced cancer prevention (36). In addition, the mechanisms of zinc inhibiting tumor reported in the existing literature include NF-kB signaling pathway (24, 25), Wnt-3a/β-catenin signaling pathway (61), P53 signaling pathway (62), hypoxia inducible factor-1α (HIF-1α) signaling pathway (63), apoptosis signaling pathway (64), and extracellular signal-regulated kinase 1/2 (ERK1/2) signaling pathway (65), etc. In a word, zinc could inhibit tumors by affecting many different signaling pathways.

In this study, the zinc supplementation experiments confirmed that the treatment with low concentration of zinc chloride could effectively inhibit the proliferation and colonies formation of ccRCC cells. In addition, overexpression of SLC39A8 could significantly inhibit 786-O and OSRC-2 cells proliferation, invasion and migration, and zinc supplementation could satisfactorily enhance this inhibitory effect. Western blotting experiments also confirmed that SLC39A8/zinc could inhibit the EMT of ccRCC cells. Based on these results and existing literatures, we propose a possible mechanism that SLC39A8/zinc inhibit the proliferation, invasion and migration of ccRCC cells (**Figure 7C**). In brief, SLC39A8 serves to pass zinc into the cytoplasm, and intracellular zinc activate P53, ERK1/2 and apoptosis-related genes and pathways, and inhibit NF-kB, ICAM-1, AP-N, IL-8, and HIF-1α related signaling pathways by participating in a series of biological processes, finally leading to increased tumor cell death, and inhibition of EMT, tumor cell proliferation, invasion, and migration.

## Conclusion

In this study, SLC39A8 was identified as a key gene of ZIP family in ccRCC, which could be used as an effective indicator for diagnosing ccRCC and judging its prognosis. With the progression of tumor, the expression of SLC39A8 decreased progressively. The prognosis of ccRCC patients with low expression of SLC39A8 is significantly worse. Overexpression of SLC39A8 or treatment with low concentration of zinc chloride could effectively inhibit the proliferation, migration and invasion of ccRCC cells. Moreover, the inhibition effect of SLC39A8 overexpression could be enhanced by low concentration zinc supplement. Therefore, this study provides a novel understanding for the role of SLC39A8/zinc in the regulation of ccRCC progression. These findings provide a new direction and target for progressive ccRCC drug development and combination therapy strategies.

## Data Availability Statement

The original contributions presented in the study are included in the article/**Supplementary Material**. Further inquiries can be directed to the corresponding authors.

## Ethics Statement

This study was fully informed by the patients and was approved by the Human Research Ethics Committee of Huazhong University of science and Technology (Wuhan, China).

## Author Contributions

ZS, XY, and KC designed the study. LL and YH wrote the manuscript. JH and LZ polished the article. LL, YH, JH, and LZ performed the experiment. LL, YH, and XY analyzed the data. ZS and KC helped revised the manuscript. All authors contributed to the article and approved the submitted version.

## Funding

This work was supported by the National Natural Science Foundation of China (Grant no. 82002722) and the Science Foundation of Wuhan (grant no. WX20Q20).

## Conflict of Interest

The authors declare that the research was conducted in the absence of any commercial or financial relationships that could be construed as a potential conflict of interest.

## Supplementary Material

Supplementary Figure 1 The relative mRNA expression of ZIP family genes in cancer tissues and corresponding normal tissues in patients with ccRCC by using Uaclan database. **(A–N)** SLC39A1, SLC39A2, SLC39A3, SLC39A4, SLC39A5, SLC39A6, SLC39A7, SLC39A8, SLC39A9, SLC39A10, SLC39A11, SLC39A12, SLC39A13, SLC39A14. Mean differences were considered statistically significant when P < 0.05.

Supplementary Figure 2 Disease-free survival of ZIP family genes in patients with ccRCC by using GEPIA. **(A–N)** SLC39A1, SLC39A2, SLC39A3, SLC39A4, SLC39A5, SLC39A6, SLC39A7, SLC39A8, SLC39A9, SLC39A10, SLC39A11, SLC39A12, SLC39A13, SLC39A14. Mean differences were considered statistically significant when P < 0.05.

Supplementary Figure 3 The clinicopathologic significance of SLC39A8 expression in the E-MTAB-1980 cohort. **(A–D)** The expression of SLC39A8 in ccRCC patients with distant or lymph node metastasis, and in different subgroups of T stage and neoplasm histologic grade. **(E)** Overall survival analysis of SLC39A8 in ccRCC patients with the high and low groups cutoffs were 35 percent and 35 percent. The p value < 0.05 was considered to have significant differences.

Supplementary Figure 4 Expression and diagnostic value of SLC39A8 in ccRCC patients and its importance in RCC cell lines. **(A)** The diagnostic significance of SLC39A8 low expression in ccRCC patients. **(B)** The antibody-based imaging from online database The Human Protein Atlas indicated that SLC39A8 protein was down-regulated in RCC tissues. **(C)** The CERES dependence score of SLC39A8 in different RCC cell lines, score approach to 0 and −1 represent the median scores of nonessential and cell-essential genes, respectively. Mean differences were considered statistically significant when P < 0.05.
