# Supplementary figures and images for: SLC39A8/Zinc Suppresses the Progression of Clear Cell Renal Cell Carcinoma
Source: Front Oncol. 2021 Mar 25;11:651921. doi: 10.3389/fonc.2021.651921 (PMC8045709; doi:10.3389/fonc.2021.651921)

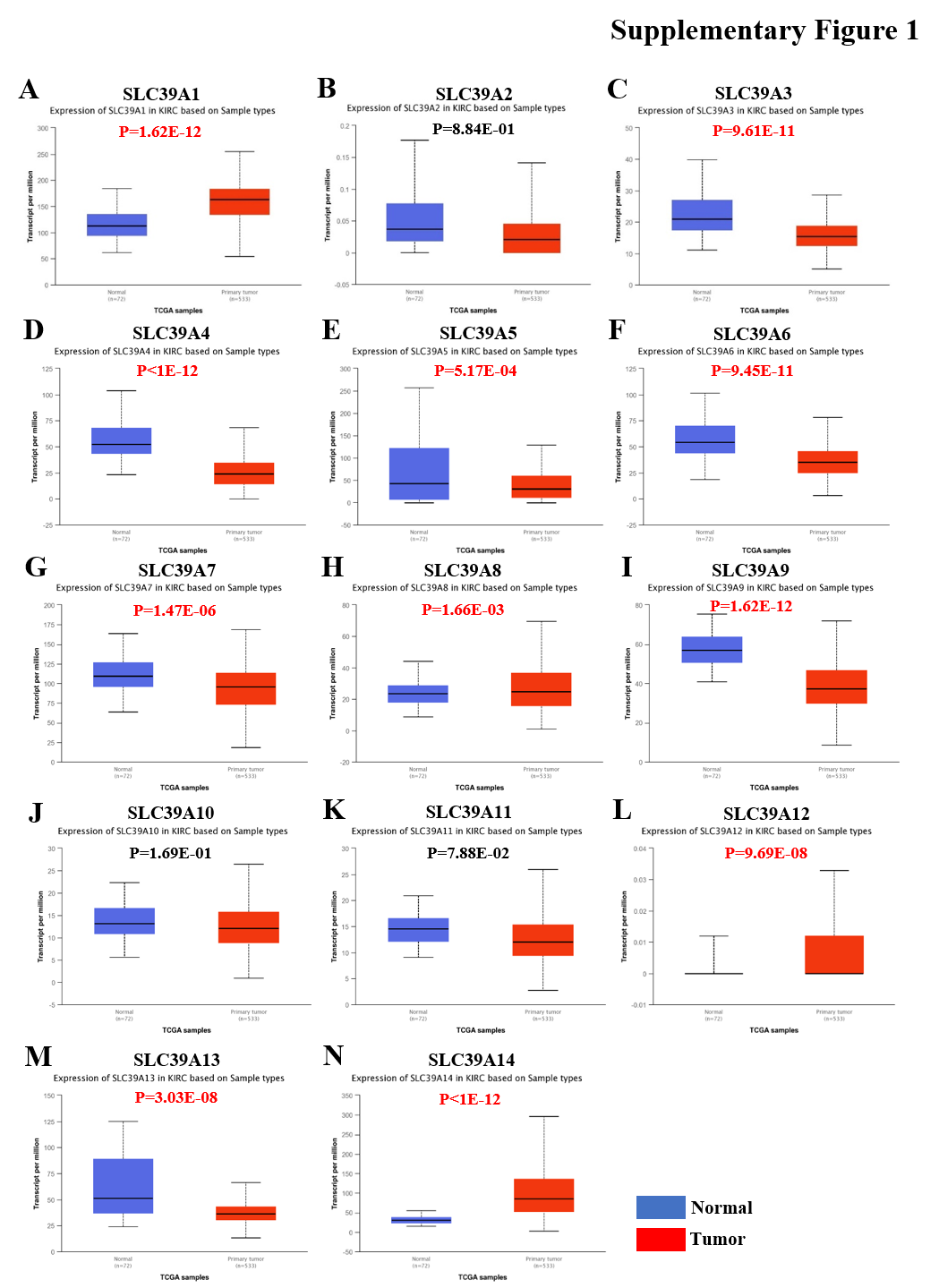

Supplement: Supplementary Figure 1 — The relative mRNA expression of ZIP family genes in cancer tissues and corresponding normal tissues in patients with ccRCC by using Uaclan database. (A–N) SLC39A1, SLC39A2, SLC39A3, SLC39A4, SLC39A5, SLC39A6, SLC39A7, SLC39A8, SLC39A9, SLC39A10, SLC39A11, SLC39A12, SLC39A13, SLC39A14. Mean differences were considered statistically significant when P < 0.05. [file Image_1.tif]

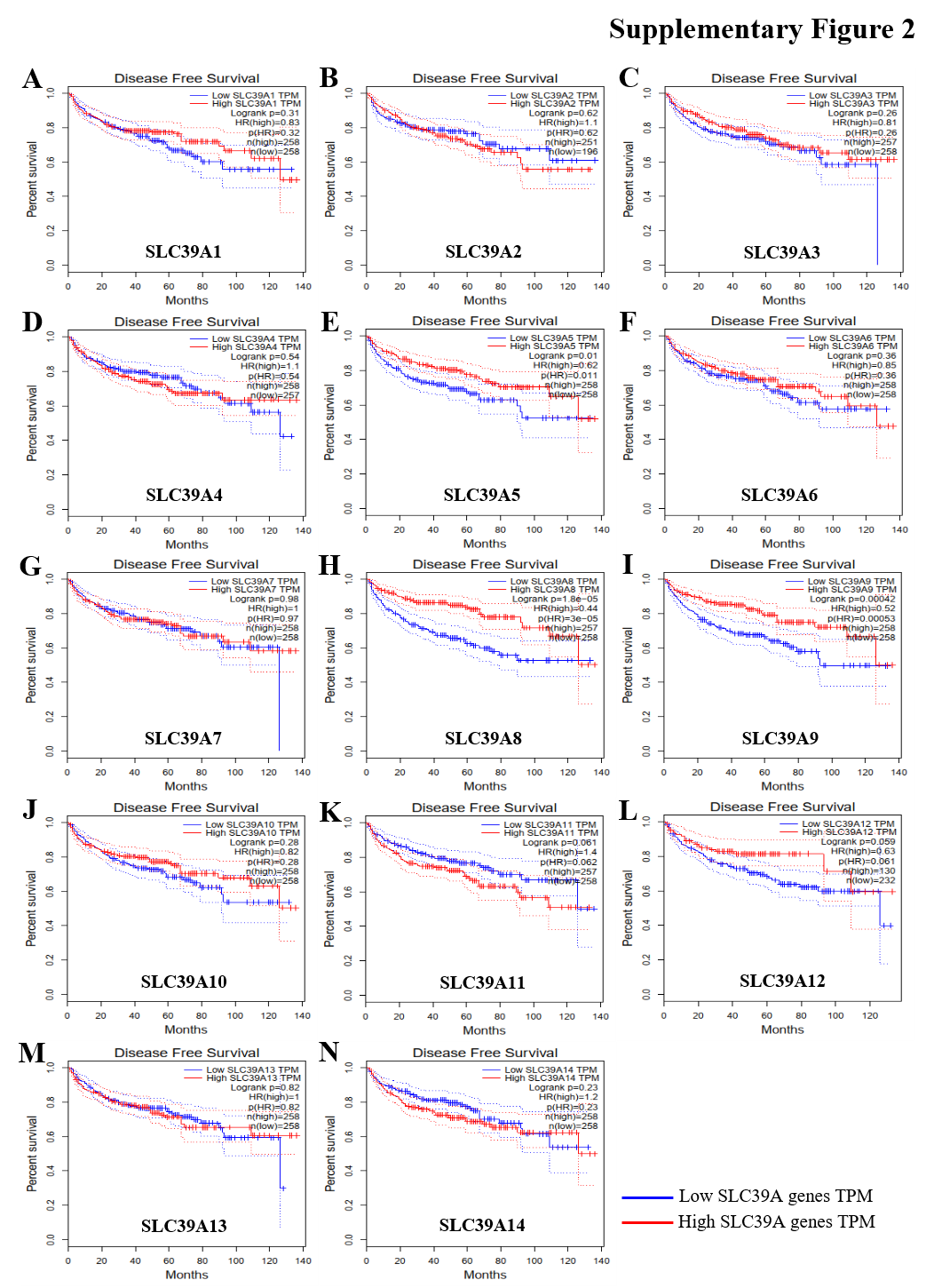

Supplement: Supplementary Figure 2 — Disease-free survival of ZIP family genes in patients with ccRCC by using GEPIA. (A–N) SLC39A1, SLC39A2, SLC39A3, SLC39A4, SLC39A5, SLC39A6, SLC39A7, SLC39A8, SLC39A9, SLC39A10, SLC39A11, SLC39A12, SLC39A13, SLC39A14. Mean differences were considered statistically significant when P < 0.05. [file Image_2.tif]

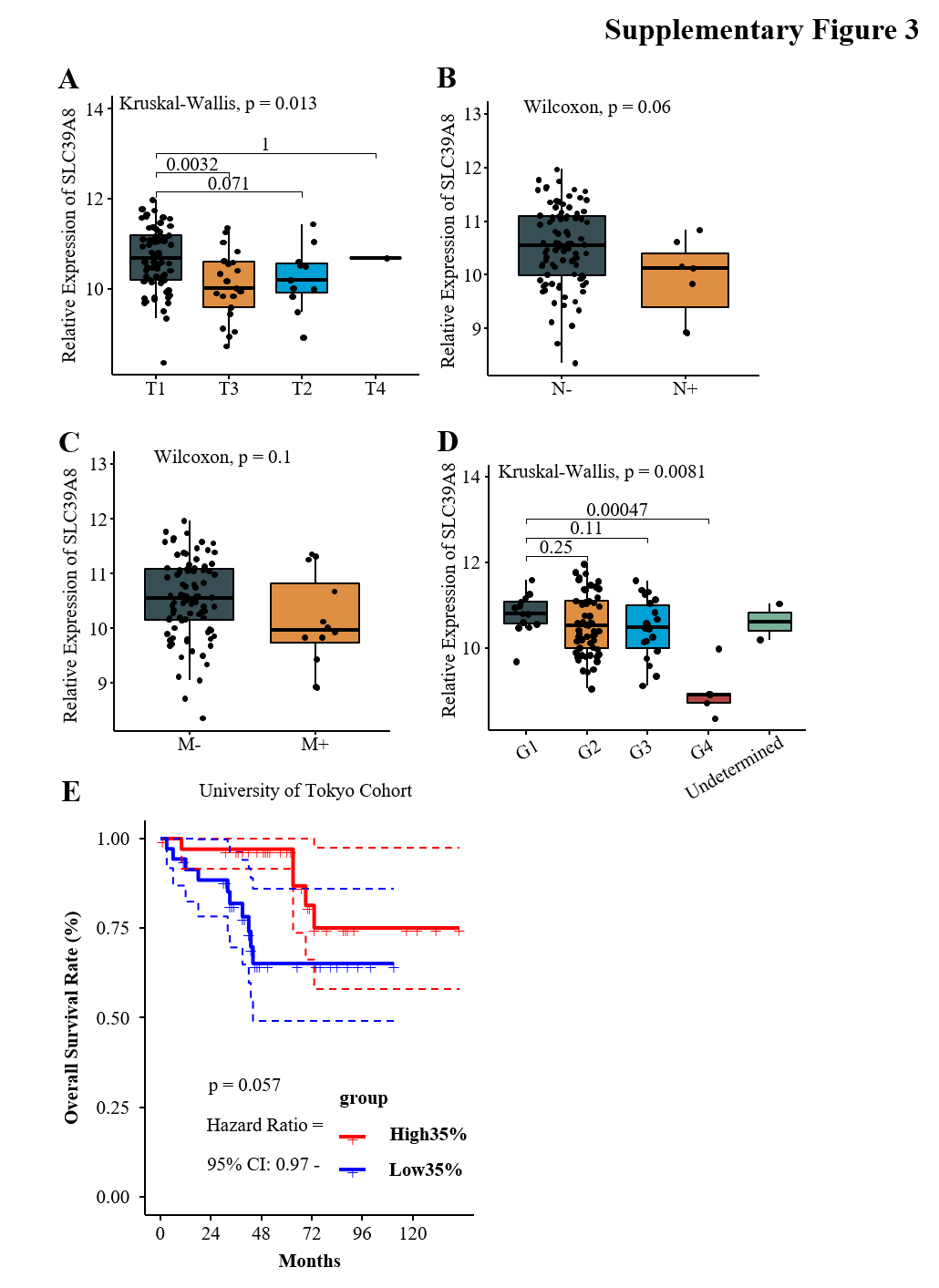

Supplement: Supplementary Figure 3 — The clinicopathologic significance of SLC39A8 expression in the E-MTAB-1980 cohort. (A–D) The expression of SLC39A8 in ccRCC patients with distant or lymph node metastasis, and in different subgroups of T stage and neoplasm histologic grade. (E) Overall survival analysis of SLC39A8 in ccRCC patients with the high and low groups cutoffs were 35 percent and 35 percent. The p value < 0.05 was considered to have significant differences. [file Image_3.tif]

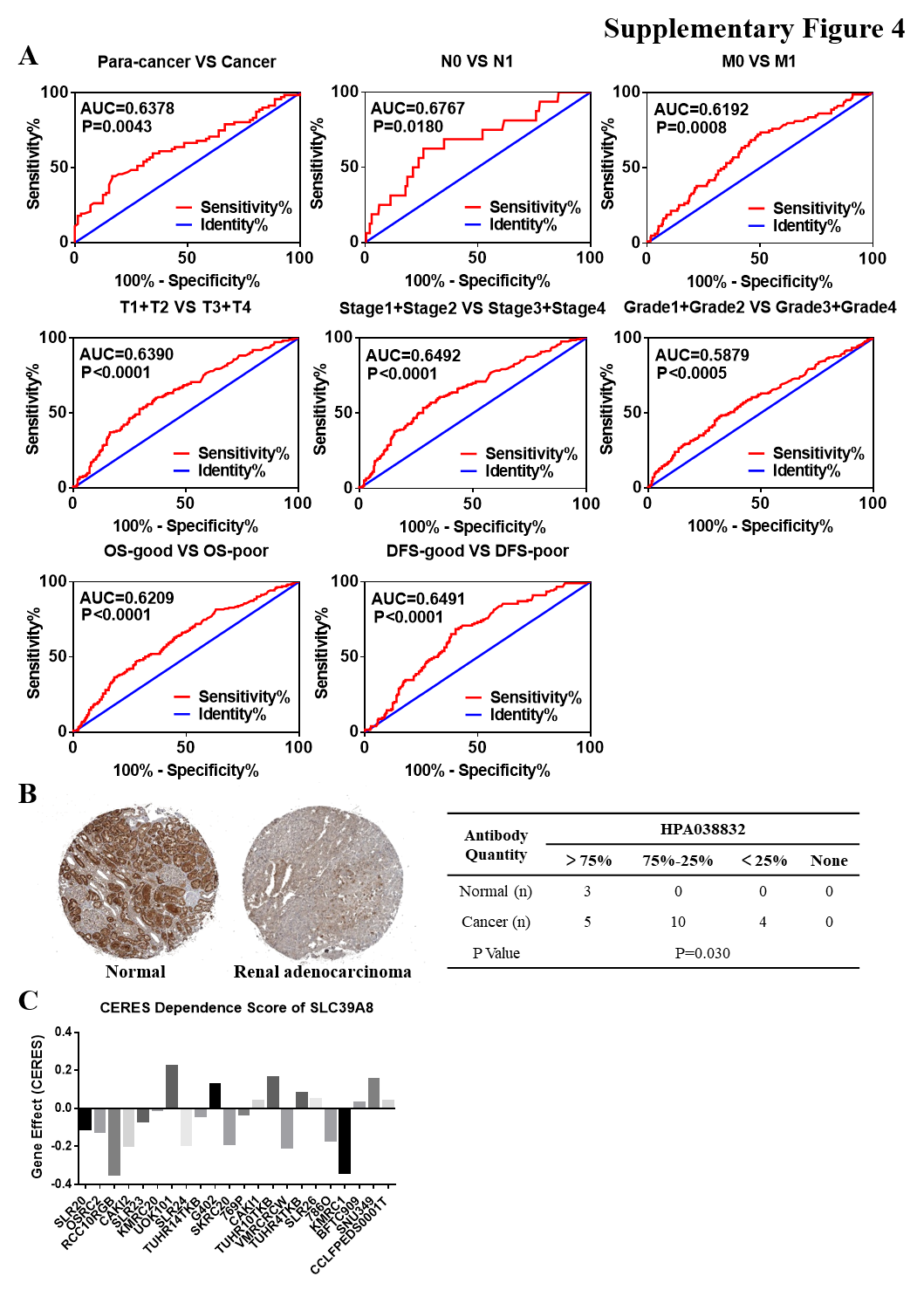

Supplement: Supplementary Figure 4 — Expression and diagnostic value of SLC39A8 in ccRCC patients and its importance in RCC cell lines. (A) The diagnostic significance of SLC39A8 low expression in ccRCC patients. (B) The antibody-based imaging from online database The Human Protein Atlas indicated that SLC39A8 protein was down-regulated in RCC tissues. (C) The CERES dependence score of SLC39A8 in different RCC cell lines, score approach to 0 and −1 represent the median scores of nonessential and cell-essential genes, respectively. Mean differences were considered statistically significant when P < 0.05. [file Image_4.tif]
